# Notes on Preparations for the Sick

**Published:** 1900-09

**Authors:** J. Odery Symes


### Notes on Preparations for the Sick.

### THE PASTEURISATION OF MILK.

An increasing amount of evidence has recently been brought forward in support of the view that milk is a very common means of spreading various zymotic diseases, chief amongst which are epidemic diarrhœa, scarlet fever, enteric fever, diphtheria, and tuberculosis. By prolonged boiling milk may be sterilised, but there is undoubtedly a strong prejudice against boiled milk, chiefly on the ground of its unpalatableness, and partly on account of its constipating action and of its lessened nutritional value. Boiling has, therefore, come to be superseded by pasteurisation. In this process the fluid is raised and kept for a long time at a temperature sufficiently high to destroy the bacterial life present, and then rapidly cooled. In this way the taste is not altered, nor does a large amount of albuminous matter rise as a scum to the surface. The exact temperature to which the milk should be raised and the time during which it should be kept at this temperature are matters of dispute. Thus, Leeds believes milk may be rendered sterile by heating to 155° F. for six minutes; and Theobald Smith recommends a temperature of 140° F. for twenty minutes, provided no scum be allowed to form. Most of these data have been determined by experiments with the tubercle bacillus, and reliable results as to the effect upon spore-bearing organisms such as may be associated with the causation of summer diarrhœa are yet wanting. There are a variety of methods employed in pasteurising on a large scale. We have recently had the opportunity of inspecting the pasteurising plant of two large dairies in the West of England; viz., those of the London, Gloucestershire, and North Hants Dairy Co. in Bristol, and of the Bath and Somersetshire Dairy Co. Lmtd. in Bath. In the former the process is briefly as follows: The milk first flows over a heater through which a continuous stream of nearly boiling water is driven, and is thus raised to a temperature of 120° F. It then passes into a high-speed centrifuge, in which all mechanical impurities are absolutely removed. After mixing the milk and cream again in a small receiver, it passes to the pasteuriser, a tinned copper vessel heated by steam, which raises the temperature of the milk to 150° F. The milk then passes into insulated tanks, where by an arrangement of hot water and steam it is kept for twenty minutes at a temperature of 150° F. The final process consists in aerating and cooling the milk. This is done by first passing through it a current of filtered air, and then passing it over capillary refrigerators which rapidly reduce the temperature to 50° F. Throughout the whole process the milk is untouched by hand, and whilst passing over the heaters and refrigerators is carefully protected from the external air. The interior of the plant is daily sterilised by steam. We were permitted to examine the temperature of the milk at all stages, and found it well within the required limits. A bacteriological examination of the milk showed that before treatment it averaged a half to one million bacteria per cubic centimetre, whilst a series of gelatin plates, each of which was inoculated with .5 c.c. of the pasteurised milk, gave an average of 85 colonies each, or 170 per c.c. These were spore-bearing, fermentative varieties, no cocci or pathogenic bacteria being detected. A specimen of the pasteurised product submitted to the centrifuge and the sediment injected into guinea-pigs did not give rise to tuberculosis or other pathogenic symptoms.

The plant of the Bath and Somerset Dairy Co. differs from the above, the procedure being as follows: The milk passes through a fine-meshed wire strainer and a cotton-wool filter into a vertical steam-jacketed turbine pasteuriser, and is here raised to a temperature of 150° F. It then passes into receiving tanks heated by steam, and the temperature maintained at 150° F. for twenty minutes. The cooler consists of a large cylinder, over the outside of which the milk flows into channels at the base, from which it is drawn off into churns. The temperature of the cooled milk is about 50° F.

From the description given above, it will be seen that pasteurised milk is not necessarily *sterilised* milk. The pathogenic organisms, the lactic acid-forming bacilli, and most of the other bacteria are killed, but a few spore-bearing, fermentative varieties may survive. These, according to Russell, form about .25 per cent. of the total number of organisms, and as their ingestion is not prejudicial to health they may be disregarded.

With regard to the important question of the alteration in the composition of milk in the process of pasteurisation, we find that although the taste and colour is unaltered, yet on standing no layer of cream rises to the top of the milk. This, however, is not due to any loss of butter-fat or cream, but to the breaking-up of the aggregation of fat globules owing to the mechanical processes through which the milk has passed. The scum which forms on the surface of boiled milk consists of coagulable albumen, and this, which is equal in amount to about one-fifth of the total casein, is generally lost. From analyses we have seen, it would appear that in the process of pasteurisation the amount of albumin is not lessened nor is the percentage of fat reduced.

We are of opinion that the process of pasteurisation affords a complete safeguard against the risk of infectious diseases conveyed by milk.

J. Odery Symes, M.D., D.P.H.

